# ^18^F-FDG PET/CT Imaging Post Heart Transplantation Depicts High Accumulation at Sites of Previous Ventricular Assist Device Insertion

**DOI:** 10.1097/RLU.0000000000004580

**Published:** 2023-02-01

**Authors:** Yoshitaka Toyama, Masayuki Otani, Nobuhiro Yaoita, Kentaro Takanami, Kei Takase

**Affiliations:** From the ∗ Department of Diagnostic Radiology, Tohoku University Hospital, Sendai, Japan; † Department of Cardiothoracic Surgery, Tohoku University Hospital, Sendai, Japan; ‡ Department of Cardiovascular Medicine, Tohoku University Graduate School of Medicine, Sendai, Japan.

**Keywords:** ^18^F-FDG PET/CT, left ventricular assist device, cardiopulmonary bypass, extracorporeal circulation

## Abstract

A 37-year-old man with previous heart transplantation for dilated cardiomyopathy underwent screening for malignancy under posttransplantation immunosuppression. ^18^F-FDG PET/CT revealed uptake in 2 peritoneal sites of the pericardium that corresponded to the insertion sites of a left ventricular assist device that was used before transplantation. Additional abnormal uptake in the right axillary artery, aortic arch, and left femoral artery corresponded to the insertion sites for arterial inflow during cardiopulmonary bypass. Knowledge that FDG accumulation may occur at the insertion sites of an extracorporeal-circulation device enables unnecessary tests to be avoided.

**FIGURE 1:**
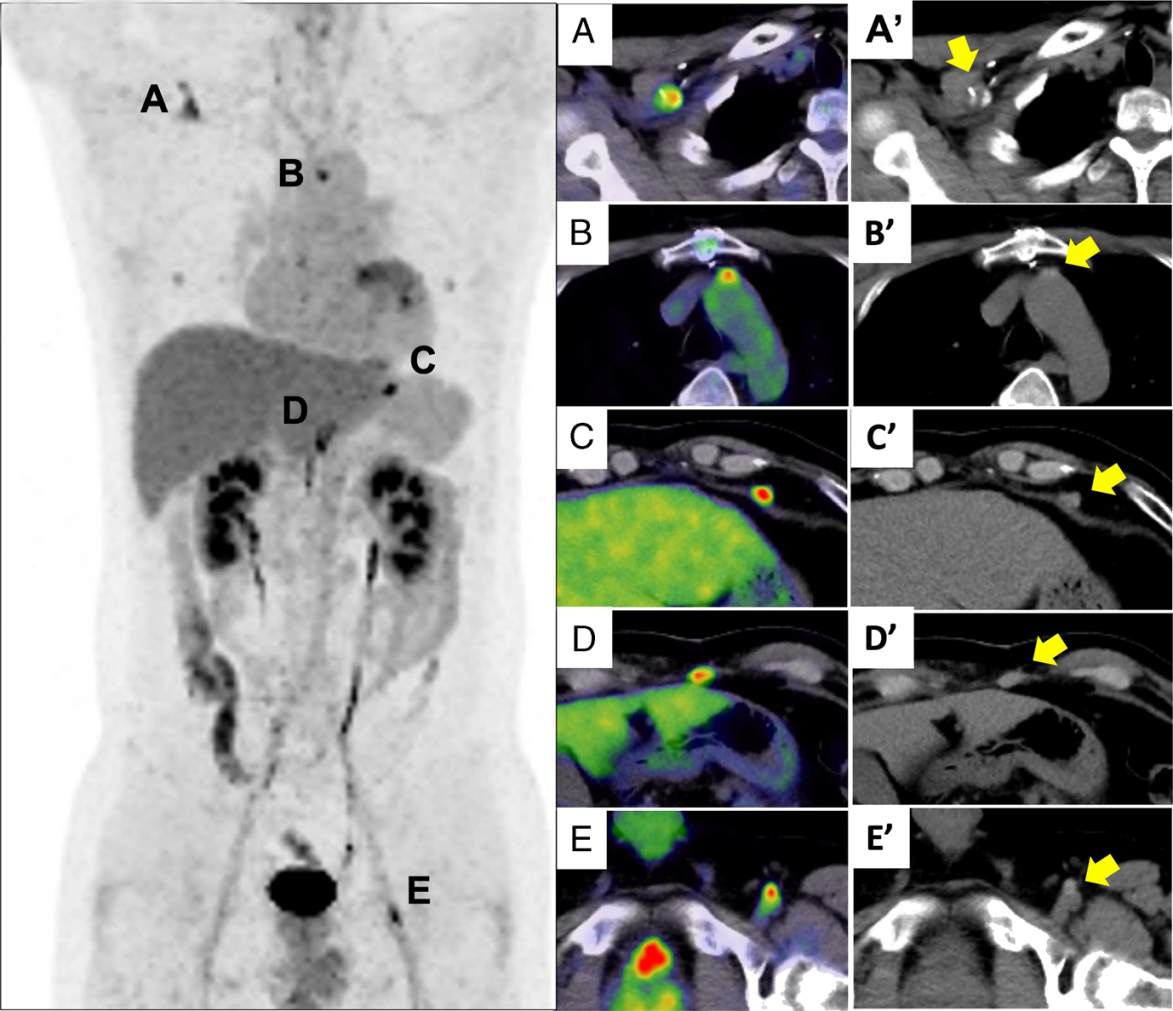
A 37-year-old man with a history of heart transplantation underwent ^18^F-FDG PET/CT in screening for malignancy under posttransplantation immunosuppression. He had no clinical symptoms and no blood data abnormalities. The screening images demonstrate FDG uptake in 2 soft tissue density nodules in the peritoneum (**C**, **D**, **C′**, and **D′**).

**FIGURE 2:**
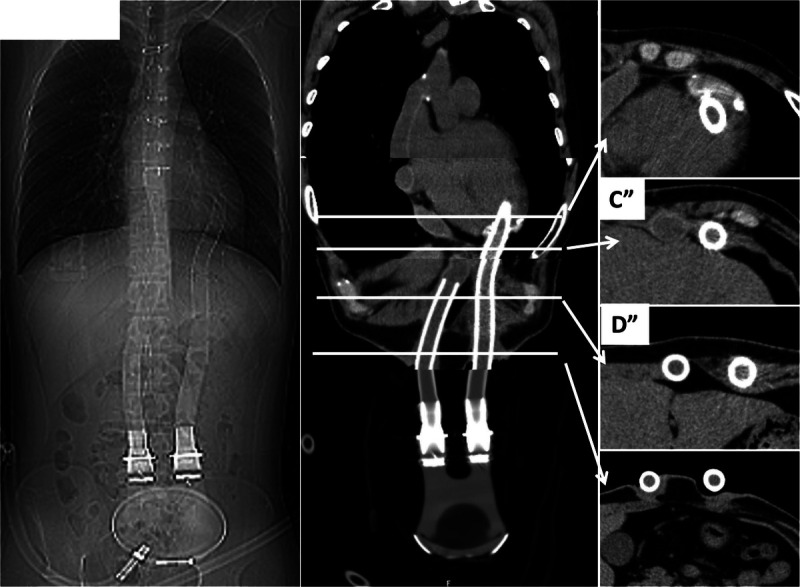
These accumulations correspond to the sites of insertion (CT scout obtained 14 years ago shows installed left ventricular assist device [LVAD] [left], reconstruction of CT coronal [middle], CT axial [right]; **C″** and **D″** correspond to the heights of **C**, **C′**, **D**, and **D′** in Fig. 1, respectively) of inflow and outflow drive lines of an LVAD used for severe dilated cardiomyopathy before heart transplantation. Abnormal uptake is also seen in high-density structures in the right axillary artery, aortic arch, and left femoral artery on CT (Fig. 1: **A**, **A′**, **B′**, **B**, **E**, and **E′**), indicating accumulation in prostheses retained at the inflow insertion site during use of the extracorporeal circulation device. According to the International Society for Heart and Lung Transplantation registry, malignancy is the most common cause of death at 5 years after transplantation, accounting for more than 20%.^1^ The most common malignancies are skin cancers followed by posttransplant lymphoproliferative disorders (PTLDs).^1,2^ According to the systematic review and meta-analysis of Montes de Jesus et al, ^18^F-FDG PET/CT is currently the imaging modality most frequently used in PTLD patients and is useful for accurate diagnosis, staging, and biopsy site selection.^3^ Another meta-analysis reported high sensitivity and specificity of FDG PET for the diagnosis of PTLD (89.7% [95% confidence interval, 84.6–93.2] and 90.9% [85.9–94.3%], respectively),^4^ but false-positives due to factors such as infection and inflammation, including healing surgical scar, should be considered.^4–6^ FDG PET/CT is also a valuable tool for evaluating device-associated infections in patients with LVAD and has high sensitivity,^7^ but to the best of our knowledge, there are no reports of abnormal uptake at the site of insertion on ^18^F-FDG PET/CT after LVAD weaning and heart transplantation. In the present case, FDG uptake occurred at multiple subcutaneous sites. FDG uptake in surgical scars has been noted since the early days of FDG-PET^8^; however, persistent FDG uptake may be due to keloid formation or a foreign body response such as stitch granuloma.^9,10^

**FIGURE 3:**
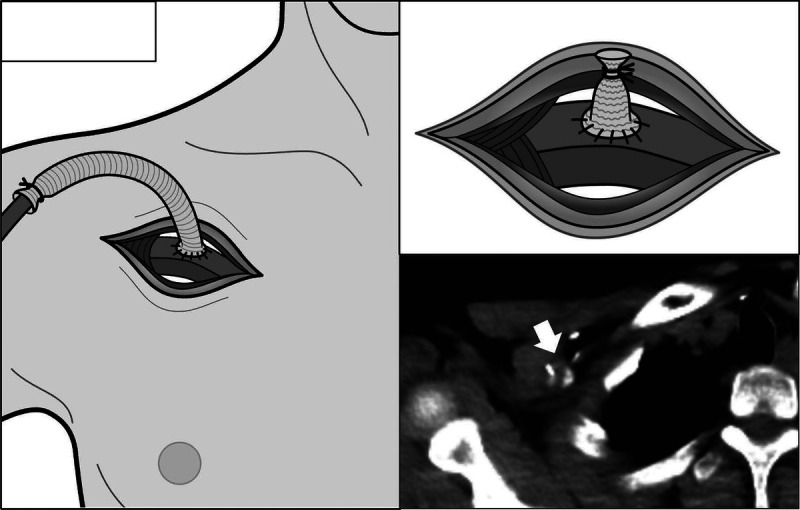
A prosthesis is sewn into insertion sites of the extracorporeal-circulation device, in the axillary or femoral artery (left); when the device is weaned, the root of the graft is ligated and the device side is excised (right). Mild-to-moderate FDG uptake has been reported even in noninfected vascular grafts.^11^ The present case was not confirmed pathologically, but was diagnosed as nonspecific FDG uptake in areas of scarring and prostheses consistent with the device insertion route more than 10 years ago. To properly diagnose malignant disease on FDG PET/CT, including PTLD after cardiac transplantation, it is necessary to know that FDG uptake may occur at the site of insertion of any previously inserted device.
